# Arthroscopic-assisted core decompression for avascular necrosis of the femoral head demonstrates favorable clinical outcomes: a systematic review

**DOI:** 10.1093/jhps/hnaf018

**Published:** 2025-04-11

**Authors:** Muzammil Akhta, Daniel Razick, Noorhan Amani, Sonia Aamer, Jimmy Wen, Trevor Shelton, Dean Wang

**Affiliations:** Orthopaedic Surgery, College of Medicine, California Northstate University, 9700 W Taron Dr, Elk Grove, CA 95757, USA; Orthopaedic Surgery, College of Medicine, California Northstate University, 9700 W Taron Dr, Elk Grove, CA 95757, USA; Orthopaedic Surgery, College of Medicine, California Northstate University, 9700 W Taron Dr, Elk Grove, CA 95757, USA; Orthopaedic Surgery, College of Medicine, California Northstate University, 9700 W Taron Dr, Elk Grove, CA 95757, USA; Orthopaedic Surgery, College of Medicine, California Northstate University, 9700 W Taron Dr, Elk Grove, CA 95757, USA; Utah Valley Orthopaedics, Provo, 1157 North 300 West, Provo, UT 84604, USA; Department of Orthopaedic Surgery, University of California, Irvine, 101 The City Drive South, Pavilion 3, Building 29A, Orange, CA 92868, USA; Department of Biomedical Engineering, University of California Irvine, 5200 Engineering Hall, Irvine, CA 92697, USA

## Abstract

This systematic review aims to evaluate clinical outcomes for arthroscopic-assisted core decompression (AACD) for avascular necrosis (AVN) of the femoral head. A literature search following Preferred Reporting Items for Systematic Reviews and Meta-Analyses guidelines was performed in PubMed, Embase, and Scopus. Nine studies were included, five comparing AACD with isolated core decompression (CD) and four evaluating outcomes of only AACD. A total of 358 patients (462 hips, 71.8% male) underwent AACD. In the five comparative studies, the AACD and isolated CD groups had 97.6% (72.2–100.0%) and 98.5% (81.0–100.0%) of hips with precollapse AVN, respectively. The modified Harris hip score, reported in five comparative studies, was significantly higher in the AACD group in four studies and not significantly different in one study. The visual analog scale pain score, reported in two comparative studies, was significantly lower in the AACD group in one study and not significantly different in the other study. The collapse rate ranged from 2.9% to 14.0% at a mean follow-up of 37.9 months in the AACD group and from 14.6% to 28.6% at a mean follow-up of 34.7 months in the isolated CD group, with all five comparative studies reporting significantly higher collapse rates in the isolated CD group. In the four AACD only studies, 42.9–100.0% of hips had precollapse AVN with the collapse rate ranging from 23.2% to 45.5% at a mean follow-up of 39.2 months. Patients undergoing AACD for treatment of AVN of the femoral head demonstrate excellent patient-reported outcomes and low rate of collapse and complications, with a possibility of superior outcomes compared to isolated CD.

## Introduction

Avascular necrosis (AVN) of the femoral head is an aseptic osteonecrosis caused by a disrupted blood supply to the proximal femur, resulting in eventual subchondral collapse of the weightbearing portions of the femoral head [1]. With an incidence of 10 000 to 20 000 cases in the USA, hip AVN commonly develops due to etiological factors including fractures, hip joint dislocations, systemic lupus erythematosus, alcohol abuse, and corticosteroids, although many cases are idiopathic [2–4]. While there is no clear consensus at this time regarding the optimal nonsurgical and surgical treatments for precollapse AVN of the femoral head, current nonsurgical treatment options include bisphosphonates, statins, and anticoagulants [5–7], although surgical management primarily consists of core decompression (CD) [8].

By drilling into the femoral head and reducing intraosseous pressure, CD aims to restore osseous circulation to the femoral head and allow for angiogenesis to occur [9]. While CD has historically been conducted percutaneously through the lateral femur, recent techniques have described utilizing arthroscopy to allow for concomitant treatment of any intra-articular pathology, more accurate diagnostic assessment for any subchondral collapse of the femoral head, and reduced risk of intra-articular penetration or bone graft extravasation [10–12]. Serong *et al*. [13] reported that in an arthroscopic evaluation of 27 hips with AVN, co-existing intra-articular pathologies were present in 96.3% of hips, including cam deformities (82%), labral defects (85%), and chondral damage (74%). Wang *et al*. [14] reported that in a series of 38 hips with precollapse AVN of the femoral head, 74% had labrum tears and 50% had significant synovitis and effusion. Concomitant arthroscopy with CD, or arthroscopic-assisted core decompression (AACD), can therefore allow for the combined management of hip AVN and other intra-articular pathologies in a single-staged procedure to treat all possible sources of hip pain. The purpose of this systematic review was to evaluate patient-reported outcomes (PROs) and survivorship of AACD for the management of hip AVN. A secondary aim was to compare the outcomes of AACD to isolated CD, when possible. The hypothesis was that AACD has good to excellent improvement of PROs and high rates of survivorship.

## Materials and methods

### Search strategy

A search following guidelines established by PRISMA (Preferred Reporting Items for Systematic Reviews and Meta-analyses) was performed on 24 June 2024, in three databases: PubMed, Embase, and Scopus. The following search terms were used to perform the systematic review: (osteonecrosis OR necrosis) AND (hip OR femur OR femoral) AND (arthroscop*). Studies were included if they reported on outcomes of AACD for management of AVN or if they compared AACD versus isolated CD. The exclusion criteria included case reports, review articles, cadaveric studies, technique articles, studies performed in animals, articles not in English, and expert opinions.

### Study selection

Two independent reviewers reviewed studies according to the eligibility criteria from the initial database search. If they were not unanimous in their decision, a third reviewer was consulted to determine study inclusion or exclusion. All included articles underwent a rigorous search of their reference lists to determine whether additional studies fit our inclusion criteria and could be added to the systematic review.

### Data extraction

Study variables extracted from the studies included authors, level of evidence (LOE), journal, study design, publication year, study period, patient inclusion and exclusion criteria, number of patients, patient age, follow-up duration, etiology of AVN, severity of AVN, additional procedures performed, preoperative and postoperative PROs, complications, failures, and rates of femoral head collapse. All extracted data were compiled for analysis using Microsoft Word (Microsoft Office 2011; Microsoft, Redmond, WA, USA).

### Quality assessment

Methodological quality was assessed using the methodological index for nonrandomized studies (MINORS) score. Two authors scored each article in the systematic review. Each author scored the article individually before the authors reviewed their scores, and any discrepancies were resolved by re-reviewing the articles until a unanimous consensus was met.

### Statistical analysis

Descriptive statistics such as means, percentages, standard deviations, ranges, and medians are reported in this review when applicable and when provided by the individual studies. A *P* < .05 was considered statistically significant. Weighted means and standard deviations were calculated using R (Version 4.3.0) with the “‘metafor” package.

## Results

**Figure 1.**
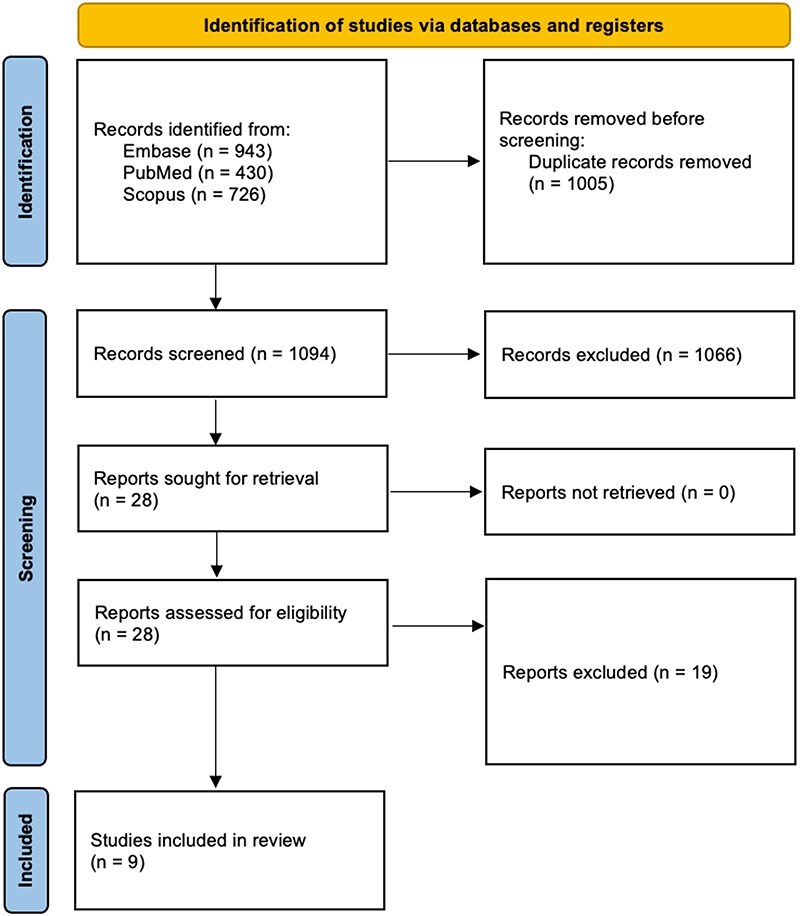
PRISMA flow diagram depicting the article selection process.

Upon the initial search, 2099 studies were identified, of which 1005 duplicates were removed. The remaining 1094 studies underwent full title and abstract screening, upon which 1066 were deemed irrelevant. The remaining 28 studies underwent full-text review, and of those, 9 were selected to be included in this systematic review [11, 15–22] (Fig. 1).

### Study characteristics and patient demographics

The studies included in this review were published between 2013 and 2024. Two studies had a prospective design, and seven had a retrospective design. The LOE was II in one study, III in four studies, and IV in four studies. Five studies compared outcomes of AACD versus isolated CD, whereas four only reported on outcomes of AACD. The mean ± standard deviation (range) of the MINORS score for comparative (five) and noncomparative (four) studies was 19.4 ± 1.5 [18–22] and 11.0 ± 1.9 [8–13], respectively. Five studies performed standard CD with a single large diameter tunnel, whereas four studies performed decompression using multiple small diameter drilling. Among the included studies, there were a total of 358 patients comprising 462 hips (71.8% male and 28.2% female) who underwent AACD. The mean ages of patients ranged from 32.7 to 44.0 years and mean follow-up times from 12.3 to 128.4 months. Study characteristics, patient demographics, and adjunct procedures are reported in Table 1.

**Table 1.** Study characteristics and patient demographics.

AACD versus isolated CD studies												
		Decompression			Number of patients/hips (M/F)		Age (years)		Follow-up duration (months)		Adjunct procedures	
Author	Study design	technique	LOE	MINORS	AACD	Isolated CD	AACD	Isolated CD	AACD	Isolated CD	AACD	Isolated CD
Zhao *et al*. [15]	Retrospective cohort	Multiple small diameter drilling	III	20/24	41/59 (25/16)	60/80 (34/26)	35.5 ± 9.8	37.7 ± 10.5	Minimum 24		Synovectomy and capsulotomy (100%)	None
Guo *et al*. [16]	Prospective randomized	Single large diameter tunnel	II	22/24	35/35 (27/8)	41/41 (29/12)	44.0 ± 6.8 ([24–55])		30		Bone grafting combined with selective arterial infusion (100%)	Selective arterial infusion (100%)
Li *et al*. [17]	Retrospective cohort	Multiple small diameter drilling	III	18/24	26/43 (15/11)	34/55 (18/16)	37.4 ± 10.3 ([21–55])	35.2 ± 11.8 ([25–32])	61.4 ± 5.7 ([21–108])	53.9 ± 4.1 ([17–89])	Labral repair/resection, cartilage debridement, synovectomy, chondroplasty, and microfracture as needed	None
Li *et al*. [18]	Retrospective cohort	Multiple small diameter drilling	III	18/24	39/53 (26/13)	52/74 (32/20)	32.7 ([20–55])	31.3 ([18–52])	39.3 ([24–52])	34.6 ([24–48])	Synovectomy (100%)	None
Yang *et al*. [19]	Retrospective cohort	Single large diameter tunnel	III	19/24	18/18 (16/2)	21/21 (18/3)	39.7 ± 8.5	40.8 ± 10.2	38.3 ± 18.9 ([24–90])	34.6 ± 8.2 ([24–55])	Bone grafting and synovectomy (100%)	Bone grafting (100%)
**AACD only studies**												
		**Decompression**			**Number of**				**Follow-up Duration**			
**Author**	**Study design**	**technique**	**LOE**	**MINORS**	**Patients/Hips (M/F)**		**Age (years)**		**(months)**		**Adjunct Procedures**	
Nazal *et al*. [11]	Retrospective case series	Single large diameter tunnel	IV	12/16	8/11 (6/2)		36.4 ± 9.2 ([17–48])		84.7 ± 21.2 (64–118)		Microfracture (45.5%), synovectomy (18.2%), labral debridement (18.2%), labral repair (18.2%), acetabular/femoral osteoplasty (18.2%)	
Ji *et al*. [20]	Prospective case series	Multiple small diameter drilling	IV	13/16	126/185 (95/31)		38.0 ([17–58])		128.4 ± 40.8 (108–144)		Synovectomy (100%)	
Ellenrieder *et al*. [21]	Retrospective case series	Single large diameter tunnel	IV	8/16	53/56 (39/14)		44.0 ± 8.2 ([18–57])		33.0 ± 20.0 ([12–74])		Bone grafting (100%) and chondral debridement or microfracture as needed	
Mutlu et al. [22]	Retrospective case series	Single large diameter tunnel	IV	11/16	12/12 (8/4)		37.3 ± 9.6		12.3 ± 4.0		Bone grafting and synovectomy (100%)	

Age, BMI, and follow-up duration are reported as mean ± standard deviation (range) when possible.

BMI, body mass index; M/F, male/female; NR, not reported.

### Etiology and severity of avascular necrosis

Six of the nine studies reported the etiology of AVN, with cited causes including alcohol use, steroid use, tobacco use, and idiopathic. Three studies comparing AACD and isolated CD reported that the etiology of AVN (alcohol use, steroid use, or idiopathic) were not significantly different between both groups. All nine studies classified the degree of AVN using either the Association Research Circulation Osseous (ARCO) (five studies), Ficat (three studies), or Steinberg (two studies) classification systems. Four studies comparing AACD and isolated CD reported that the degree of AVN was not significantly different between both groups. In the five comparative studies, the pooled percentage of patients with precollapse AVN was 97.6% (range, 72.2–100%) for the AACD group and 98.5% (range, 81.0–100%) for the isolated CD group. In the four AACD only studies, the pooled percentage of patients with precollapse AVN was 76.9% (range, 42.9–100%) (Table 2).

**Table 2.** Etiology and degree of avascular necrosis.

AACD versus isolated CD studies						
	Etiology of AVN			Severity of AVN		
Author	AACD	Isolated CD	*P*-value	AACD	Isolated CD	*P*-value
Zhao *et al*. [15]	Alcohol use: 11 (26.8%)	Alcohol use: 17 (28.3%)	.986	ARCO stage I: 18 (30.5%)	ARCO stage I: 21 (26.3%)	.581
	Steroid use: 16 (39.0%)	Steroid use: 23 (38.3%)		ARCO stage II: 41 (69.5%)	ARCO stage II: 59 (73.8%)	
	Idiopathic: 14 (34.1%)	Idiopathic: 20 (33.3%)				
Guo *et al*. [16]	NR		NR	Ficat stage I: 0 (0.0%)	Ficat stage I: 0 (0.0%)	.999
				Ficat stage II: 35 (100.0%)	Ficat stage II: 41 (100.0%)	
Li *et al*. [17]	Alcohol use: 19 (44.2%)	Alcohol use: 24 (43.6%)	0.573	ARCO stage I: 12 (27.9%)	ARCO stage I: 17 (30.9%)	>.05
	Steroid use: 15 (34.9%)	Steroid use: 18 (32.7%)		ARCO stage II: 31 (72.1%)	ARCO stage II: 38 (69.1%)	
	Idiopathic: 9 (20.9%)	Idiopathic: 13 (23.6%)				
Li *et al*. [18]	Alcohol use: 24 (61.5%)	Alcohol use: 34 (65.4%)	>.05	Ficat stage I: 15 (28.3%)	Ficat stage I: 24 (32.4%)	.062
	Steroid use: 12 (30.8%)	Steroid use: 13 (25.0%)		Ficat stage II: 38 (71.7%)	Ficat stage II: 50 (67.6%)	
	Idiopathic: 3 (7.7%)	Idiopathic: 5 (9.6%)				
Yang *et al*. [19]	NR		NR	ARCO stage I: 0	ARCO stage I: 0	>.05
				ARCO stage II: 13 (72.2%)	ARCO stage II: 17 (80.1%)	
				ARCO stage III: 5 (27.8%)	ARCO stage III: 4 (19.9%)	
**AACD only studies**						
**Author**	**Etiology of AVN**			**Severity of AVN**		
Nazal *et al*. [11]	Steroid use: 2 (33.3%)			Ficat stage I: 3 (27.3%)		
	Smoking tobacco: 4 (66.6%)			Ficat stage IIa: 4 (36.4%)		
				Ficat stage IIb: 4 (36.4%)		
Ji *et al*. [20]	Alcohol use: 53 (42.1%)			ARCO stage I: 43 (23.2%)		
	Steroid use: 48 (38.1%)			ARCO stage II: 114 (61.6%)		
	Idiopathic: 25 (19.8%)			ARCO Stage III: 28 (15.1%)		
Ellenrieder *et al*. [21]	NR			Steinberg stage I: 3 (5.4%)		
				Steinberg stage II: 21 (37.5%)		
				Steinberg stage III: 12 (21.4%)		
				Steinberg stage IV: 20 (35.7%)		
Mutlu *et al*. [22]	Steroid use: 10 (83.3%)			Steinberg stage I: 3 (25%)		
	Idiopathic: 2 (16.7%)			Steinberg stage II: 8 (66.7%)		
				Steinberg stage III: 1 (8.3%)		

NR, not reported.

### Patient-reported outcomes

Preoperatively, four comparative and two noncomparative studies reported one or more PRO measurements. The modified Harris hip score (mHHS), reported in four comparative studies, was not significantly different between the AACD and isolated CD in all studies. The visual analog scale (VAS) pain score, reported in two comparative studies, was significantly higher in the AACD group in one study and not significantly different between AACD and isolated CD in another study (Table 3).

**Table 3.** Patient-reported outcomes.

AACD versus isolated CD studies							
	Patient-reported outcomes						
		Preoperative			Postoperative		
Author	PRO	AACD	Isolated CD	*P*-value	AACD	Isolated CD	*P*-value
Zhao *et al*. [15]	mHHS	59.5 ± 11.6	57.7 ± 9.5	.3168	81.9 ± 10.2	76.4 ± 9.1	.0011
	VAS pain	6.3 ± 2.1	6.6 ± 2.5	.4561	3.1 ± 1.7	3.9 ± 2.1	.0176
Guo *et al*. [16]	mHHS	NR			86.7 ± 4.4	78.6 ± 5.6	<.05
Li *et al*. [17]	mHHS	68.2 ± 11.4	69.5 ± 9.7	.179	82.1 ± 2.9	75.8 ± 4.1	.017
Li *et al*. [18]	mHHS	66.6 ± 10.3	68.9 ± 9.2	>.05	83.3 ± 8.8	76.6 ± 9.2	.029
Yang *et al*. [19]	mHHS	59.8 ± 7.7	57.5 ± 5.8	.941	80.1 ± 9.2	75.1 ± 12.7	.112
	VAS pain	5.7 ± 0.9	4.4 ± 1.2	.004	1.2 ± 2.2	2.2 ± 2.8	.276
**AACD only studies**							
	**Patient-reported outcomes**						
**Author**	**PRO**	**Preoperative**			**Postoperative**		
Nazal *et al*. [11]	VAS pain	NR			0.8 ± 0.4 ([0–1])		
Ji *et al*. [20]	mHHS	67.1			88.3		
	VAS pain	9.1 ± 0.5			NR		
Ellenrieder *et al*. [21]	NR						
Mutlu *et al*. [22]	mHHS	41.7 ± 6.7			86.8 ± 6.6		
	VAS pain	9.2 ± 6.7			1.2 ± 0.1		

Patient-reported outcomes are reported as mean ± standard deviation (range) when possible. The *P*-values compare the postoperative patient-reported outcomes in the AACD versus isolated CD groups.

NR, not reported.

At the latest follow-up, five comparative studies and three noncomparative studies reported one or more PRO measurements. The mHHS, reported in five comparative studies, was significantly higher in the AACD group in four studies and not significantly different between AACD and isolated CD in one study. The VAS pain score, reported in two comparative studies, was significantly lower in the AACD group in one study and not significantly different between AACD and isolated CD in another study (Table 3). In studies comparing AACD and isolated CD, the weighted mean ± standard deviation (range) of the postoperative mHHS score was 85.5 ± 8.0 (80.1–88.3) for AACD and 76.6 ± 8.2 (75.1–78.6) for isolated CD. Additionally, in studies comparing AACD and isolated CD, the weighted mean ± standard deviation (range) of the postoperative VAS pain score was 2.3 ± 1.9 (0.8–3.1) for AACD and 3.5 ± 2.4 (2.2–3.9) for isolated CD.

Whether or not there were significant preoperative to latest follow-up improvements in PROs was reported in three comparative studies [15, 17, 18] and two noncomparative studies [20, 22]. All three comparative studies reported significant improvements in the mHHS for both AACD and isolated CD. Only one of these three comparative studies also reported the VAS pain score and found significant improvements for AACD and isolated CD [15]. Both noncomparative studies reported significant improvements in all the mHHS for AACD. Only one of the two noncomparative studies also reported the VAS pain score and found a significant improvement for AACD [22].

### Femoral head collapse following core decompression

All nine studies reported the incidence of femoral head collapse at follow-up in patients following CD. In the five comparative studies, the collapse rate ranged from 2.9% to 14.0% in the AACD group at a mean follow-up of 37.9 months and from 14.6% to 28.6% in the isolated CD group at a mean follow-up of 34.7 months. All of the five comparative studies reported significantly higher rates of collapse in isolated CD groups. The collapse rate in the four noncomparative studies ranged from 23.2% to 45.5% at a mean follow-up of 39.2 months (Table 4).

**Table 4.** Progression of avascular necrosis and surgical complications.

AACD versus isolated CD studies					
	Collapse rate				
	*N* (%)				
Author	AACD	Isolated CD	*P*-value	Notes	Complications
Zhao *et al*. [15]	6 (10.2)	22 (27.5)	.0352	6/6 in AACD group were ARCO stage II. 4/22 in isolated CD group were ARCO stage I and 18/22 were ARCO stage II.	None
Guo *et al*. [16]	1 (2.9)	6 (14.6)	<.05	-	NR
Li *et al*. [17]	6 (14.0)	14 (25.5)	.02	1/6 in AACD group were ARCO stage I and 5/6 were ARCO stage II. 3/14 in isolated CD group were ARCO stage I and 11/14 were ARCO stage II. Collapse rates in ARCO stage I and II were significantly higher in isolated CD group (P = 0.017 and 0.04, respectively). All collapsed patients underwent THA.	None
Li *et al*. [18]	6 (11.3)	16 (21.6)	.041	1/6 in AACD group were Ficat stage I and 5/6 were Ficat stage II. 4/16 in isolated CD group were Ficat stage I and 12/16 were Ficat stage II. Collapse rates in Ficat stage I and II were significantly higher in isolated CD group (*P* = .032 and .034, respectively). All collapsed patients underwent THA.	AACD group had 2 (3.8%) temporary sciatic nerve incompetence caused by traction. Both recovered within 2 weeks of operation.
Yang *et al*. [19]	2 (11.1)	6 (28.6)	<.05	All collapsed patients underwent THA.	None
**AACD only studies**					
	**Collapse rate**				
**Author**	***N* (%)**		**Notes**		**Complications**
Nazal *et al*. [11]	5 (45.5)		3/5 were Ficat stage I and 2/5 were Ficat stage IIa. All 5 were converted to THA at 22.6 ± 8.5 ([7–33]) months following index surgery.		None
Ji *et al*. [20]	44 (23.8)		29/44 were converted to THA, 5/44 underwent vascularized bone grafting, and 10/44 declined secondary surgery.		NR
Ellenrieder *et al*. [21]	13 (23.2)		2/13 were Steinberg stage II, 3/13 were Steinberg stage III, 4/13 were Steinberg stage IVa, and 4/13 were Steinberg stage IVb/c.		Temporary pressure lesion of pudendal nerve with complete regression in 2 patients (2.6%) and Brooker grade II heterotropic ossification as a result of hip arthroscopy in 2 patients (2.6%).
Mutlu *et al*. [22]	3 (25)		1/3 were Steinberg I and 2/3 were Steinberg II. All underwent subsequent THA		None

NR, not reported; *N*, number of patients.

### Surgical complications

Complications were reported in four of the five comparative studies. The complication rate ranged from 0% to 3.8% in the AACD group and was 0% in the isolated CD group. Three studies reported no complications in both AACD and isolated CD groups, and one reported temporary sciatic nerve damage in two patients (3.8%) in the AACD group but no complications in the isolated CD group. Three of the four noncomparative AACD only studies reported complications, with a complication rate ranging from 0% to 5.2%. Two studies reported no complications and the other reporting temporary pudendal nerve damage in two patients (2.6%) and Brooker grade II heterotopic ossification due to hip arthroscopy in two patients (2.6%) (Table 4).

## Discussion

In this systematic review of nine studies, AACD patients demonstrated excellent post-operative PROs and low complication rates. When compared to isolated CD in five studies, AACD patients had significantly higher postoperative mHHS scores in four of five studies, significantly lower VAS pain scores in one of two studies, and a significantly lower risk of collapse at final follow-up in five of five studies. Complication rates were similar between both groups though a few patients undergoing AACD did experience temporary sciatic and pudendal nerve damage. Overall, concomitant arthroscopy with CD appears to be safe and may improve hip pain and function more than traditional isolated CD.

Studies have demonstrated that AVN is often associated with the presence of additional hip pathologies such as labral tears, FAI, chondral lesions, and synovitis [13, 14, 23, 24]. In a cohort of patients with stage 2 AVN, Serong et al. [25] reported that the prevalence of cam type FAI, defined by an alpha angle of ≥ 60° or head-neck offset of ≤ 9 mm, was 47.7% and 90.7%, respectively. They then evaluated outcomes of a modified CD technique with a new percutaneous expandable reamer and found that of 32 patients who experienced treatment failure, defined as conversion to THA, the rate of failure was 40.7% in patients with an alpha angle of ≥ 60° compared to 27.7% in patients with an alpha angle of < 60°. With concomitant hip pathologies being commonly present with AVN, isolated CD may not be sufficient in addressing all possible sources of hip pain. The addition of arthroscopy allows for a more thorough diagnostic assessment for any subchondral collapse of the femoral head, reduced risk of intra-articular penetration or bone graft extravasation, and concomitant arthroscopic treatment of hip pathologies [9, 10, 26, 27]. Additionally, during the treatment of intra-articular pathologies, capsulotomies are often performed arthroscopically, which may indirectly reduce intraosseous pressure and increase perfusion, although this has only been proven in the setting of hemarthrosis from fractures. Further studies are warranted regarding whether arthroscopic interventions allow for superior outcomes in the treatment of hip AVN, as suggested by the results of the present study.

A noticeable finding from two of the included studies [17, 18] was that while AACD versus isolated CD patients with stage 1 AVN did not have significant differences in postoperative PROs, postoperative PROs were significantly better in the AACD group compared to the isolated CD group in patients with stage 2 AVN. Additionally in both studies, collapse rates were significantly lower in both AVN stage 1 and 2 AACD groups. Wang et al. [14] showed that the severity of AVN correlated with a higher rate of labrum tears and synovitis. This further suggests that the addition of arthroscopy may confer superior diagnostic and therapeutic benefits compared to isolated CD, especially in patients with more advanced grades of AVN.

While patient-acceptable symptomatic state (PASS) values for the mHHS have not been established for patients undergoing AACD or isolated CD, a recent systematic review that compiled the PASS values for patients undergoing hip arthroscopy for femoroacetabular impingement found that the across 11 studies at mean follow-up times ranging from 12.0 to 120.0 months, the PASS values for the mHHS ranged from 74.0 to 97.0 [28]. Based on those extrapolated values, in both studies by Li et al. [17, 18] in our systematic review, the PASS was achieved for patients undergoing both AACD (84.6 and 86.2) or isolated CD (83.6 and 84.6) for stage I AVN. However, for stage II AVN, in both studies only the AACD groups successfully achieved the PASS (80.0 and 79.4) whereas the isolated CD groups did not (71.4 and 72.1).

Four of the nine studies performed the CD by drilling multiple small diameter tunnels rather than creating a single large diameter tunnel. When comparing the multiple small diameter drilling versus single large diameter tunnel studies in this meta-analysis, the pooled mean postoperative mHHS (85.6 versus 84.9) and pooled collapse rates (23.5% versus 25%) were comparable between group. A biomechanical study found that when comparing multiple small 3 mm tunnels with one large 8 mm tunnel, the multiple tunnel group withstood a significantly greater load prior to failure (P < 0.05), the single large tunnel group removed a significantly greater volume of bone (P < 0.001), and both groups has a similar total fracture energy (P > 0.05) [29]. Mont et al. [30] reported in a prospective study in 2004 that multiple small diameter tunnels had a 71.0% survivorship at a follow-up of two years along with minimal morbidity with no serious complications such as subtrochanteric fractures, which have historically been associated with decompression using a single large diameter tunnel. The advent of expandable reamers allows for a single small diameter tunnel while being able to expand and ream the necrotic bone in the femoral head, thereby decreasing femoral neck stress risers and risk of fracture [10].

This systematic review should be considered in the context of its limitations. First, there was substantial variability in the follow-up period between studies, ranging from 12.3 to 128.4 months, and studies with shorter follow-up periods may demonstrate lower collapse rates. Second, the included studies varied in their classification system used to determine the severity of AVN, which precluded a formal subanalysis of postoperative mHHS scores and collapse rates in patients with different severities of preoperative AVN. While three of the nine total studies compared outcomes in patients with different severities of AVN, two used the ARCO classification system, and one used the Ficat staging classification systems. Third, concomitant arthroscopic procedures such as labral repair and femoral head osteoplasty were either not reported at all or were reported with substantial heterogeneity. As a result, it is not clear whether concomitant arthroscopic treatment of labrum and cam pathologies actually improve patient outcomes, and this requires further study. Fourth, the specific technique used for CD differed between studies, with five drilling a single large diameter tunnel and four performing multiple small diameter drilling. While pooled mean mHHS and collapse rates between both techniques were comparable, a formal statistical comparison was not possible.

## Conclusions

AACD allows for simultaneous visual assessment of the femoral head and treatment of intra-articular hip pathologies during the management of AVN. Patients undergoing AACD for treatment of AVN of the femoral head demonstrated excellent PROs, a lower rate of collapse compared to isolated CD, and a low risk of complications.

## Data Availability

No new data were generated in support of this research
